# Supplementary material for: Bimetallic TiO2 Nanoparticles for Lignin-Based Model Compounds Valorization by Integrating an Optocatalytic Flow-Microreactor
Source: Molecules. 2022 Dec 9;27(24):8731. doi: 10.3390/molecules27248731 (PMC9785458; doi:10.3390/molecules27248731)
Supplement: Supplementary file 1 [file molecules-27-08731-s001.zip › molecules-2045285-supplementary.pdf]

Bimetallic TiO_2 Nanoparticles for Lignin-Based Model Compounds Valorization by Integrating Optocatalytic Flow-Microreactor

Swaraj rashmi Pradhan [1,*], Marta PaszkiewiczGawron [1], Dariusz Łomot [1], Dmytro Lisovytskiy [1] and Juan Carlos Colmenares [1,*]

[1] Institute of Physical Chemistry, Polish Academy of Sciences, Kasprzaka 44/52, 01-224 Warsaw, Poland;
* Correspondence: jcarloscolmenares@ichf.edu.pl, srpradhan@ichf.edu.pl ; Tel.: +48 22 343 32152

Supplementary Information

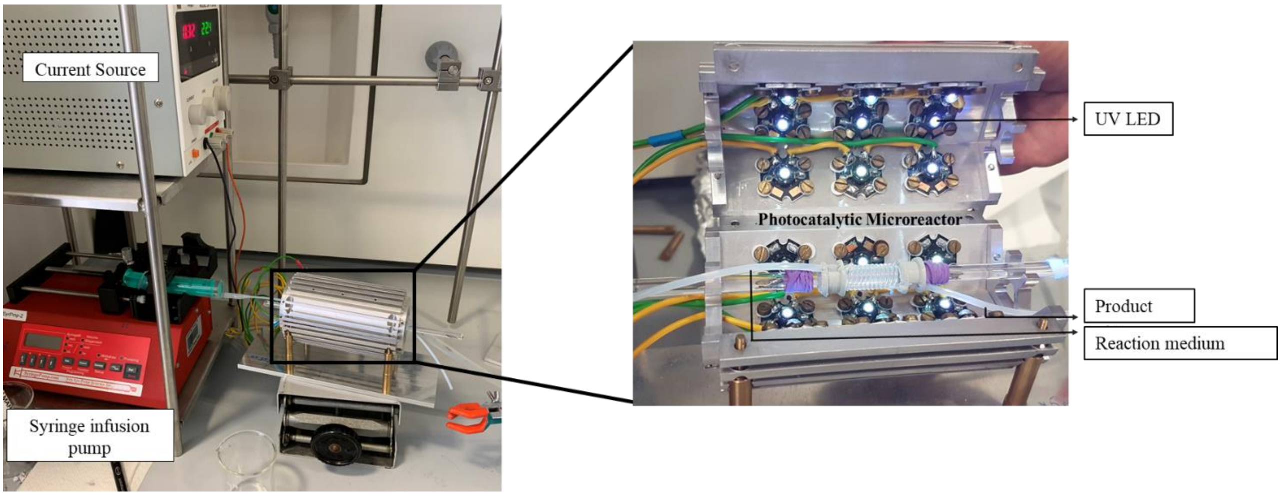

Figure S1. Experimental set up for photocatalysis in microflow system.

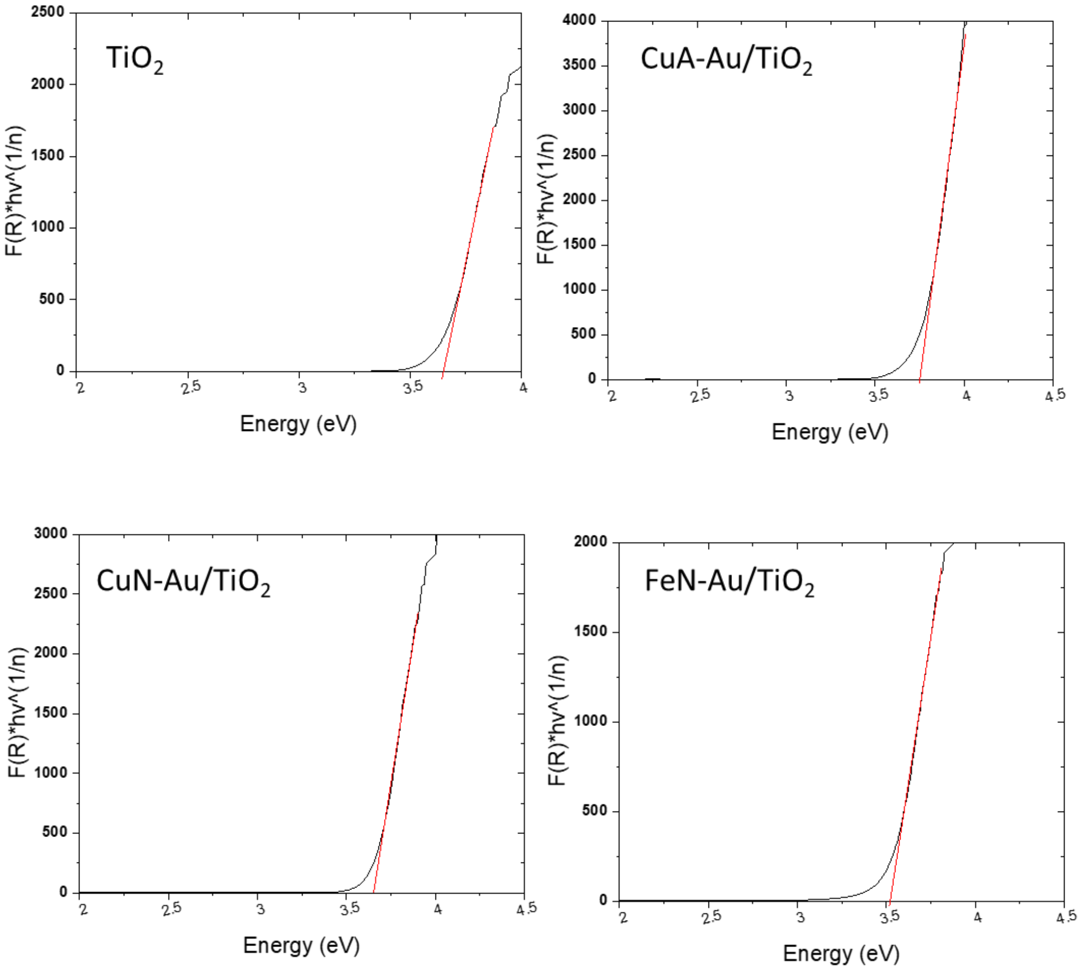

Figure S2. Bandgap calculation of sol-gel synthesized TiO_2 and bimetallic TiO_2 (CuA-Au/TiO_2, CuN-Au/TiO_2, FeN-Au/TiO_2) catalysts.

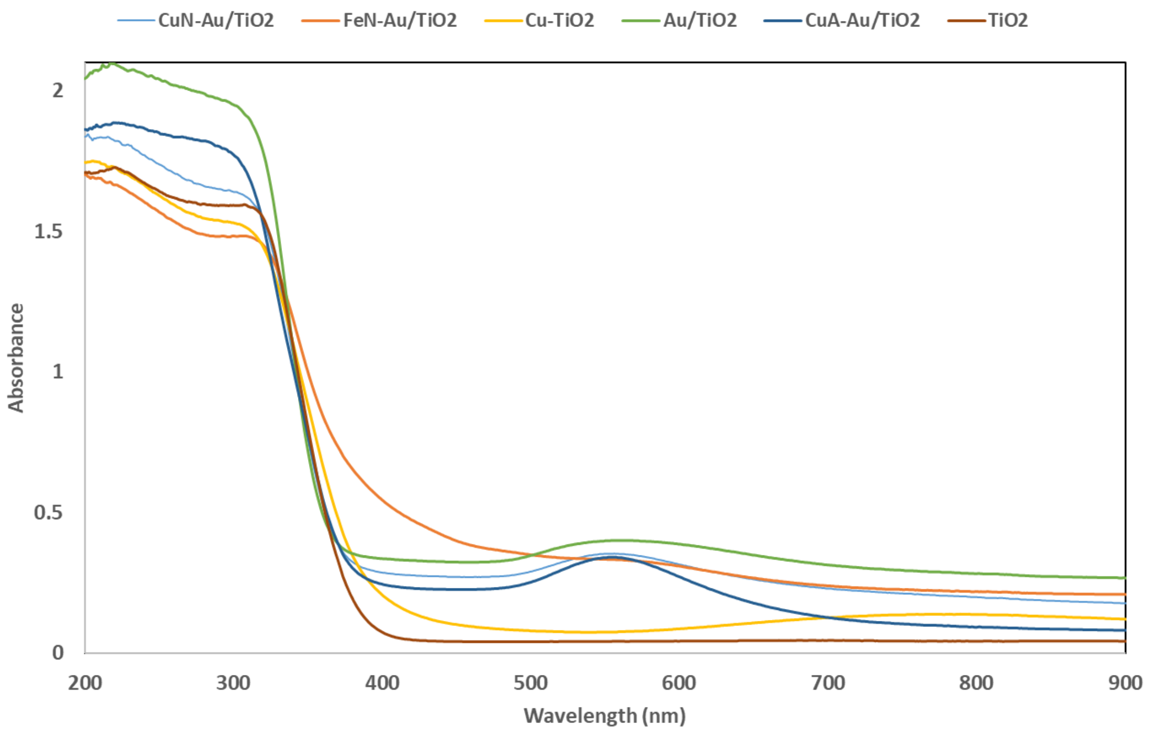

Figure S3. UV-vis diffuse reflectance spectra of synthesized bimetallic TiO_2.

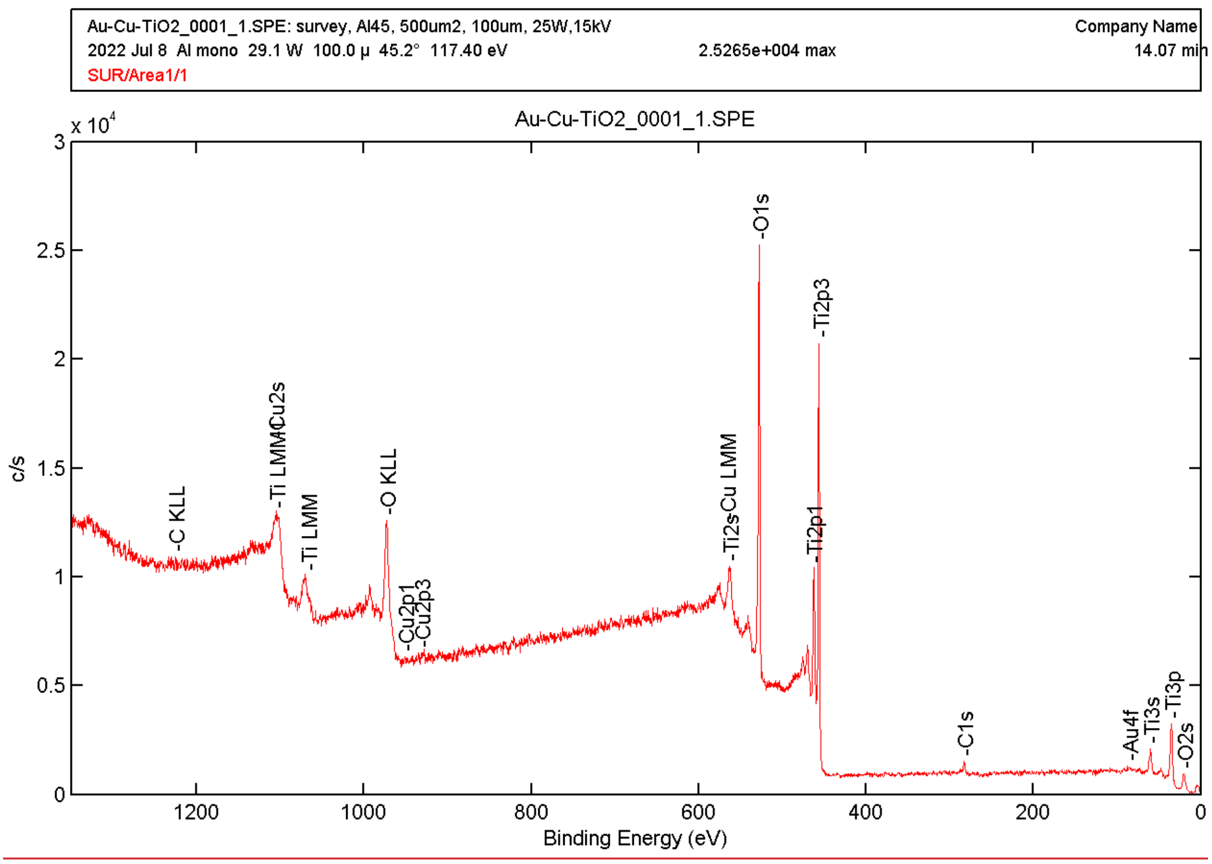
Au-Cu-TiO2_0001_1.SPE: survey, Al45, 500um2, 100um, 25W,15kV
2022 Jul 8 Al mono 29.1 W 100.0 µ 45.2° 117.40 eV
2.5265e+004 max
Company Name
14.07 min
SUR/Area1/1
Au-Cu-TiO2_0001_1.SPE
x 10^4
c/s
Binding Energy (eV)
-O1s
-Ti2p3
-Ti2p1
-Ti LMM
-Cu2s
-C KLL
-Ti LMM
-O KLL
-Cu2p1
-Cu2p3
-Ti2s
-Cu LMM
-C1s
-Au4f
-Ti3s
-Ti3p
-O2s

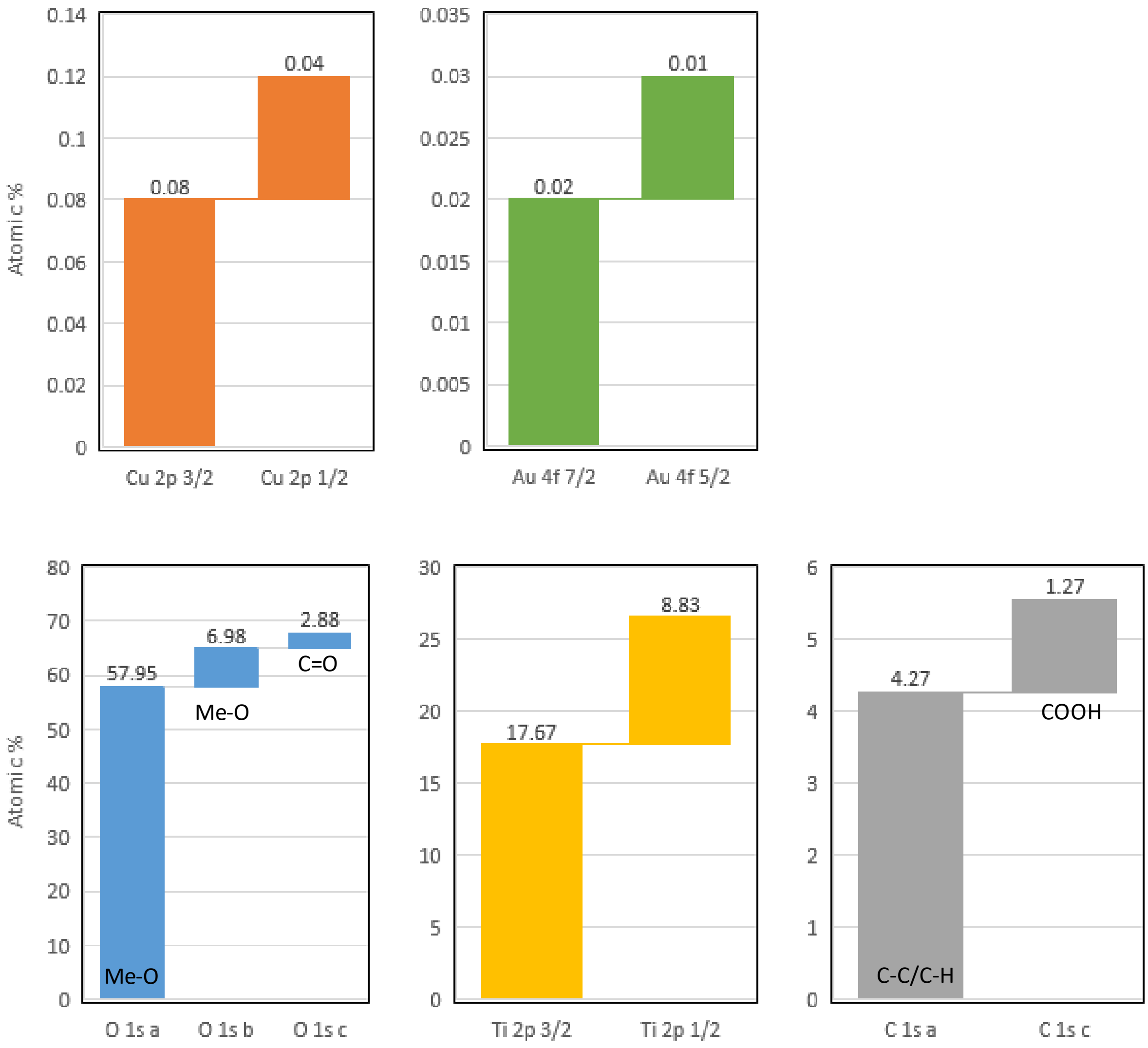

Figure S4. Surface analysis of CuA/Au-TiO_2 by X-ray photoelectron spectroscopy (XPS).

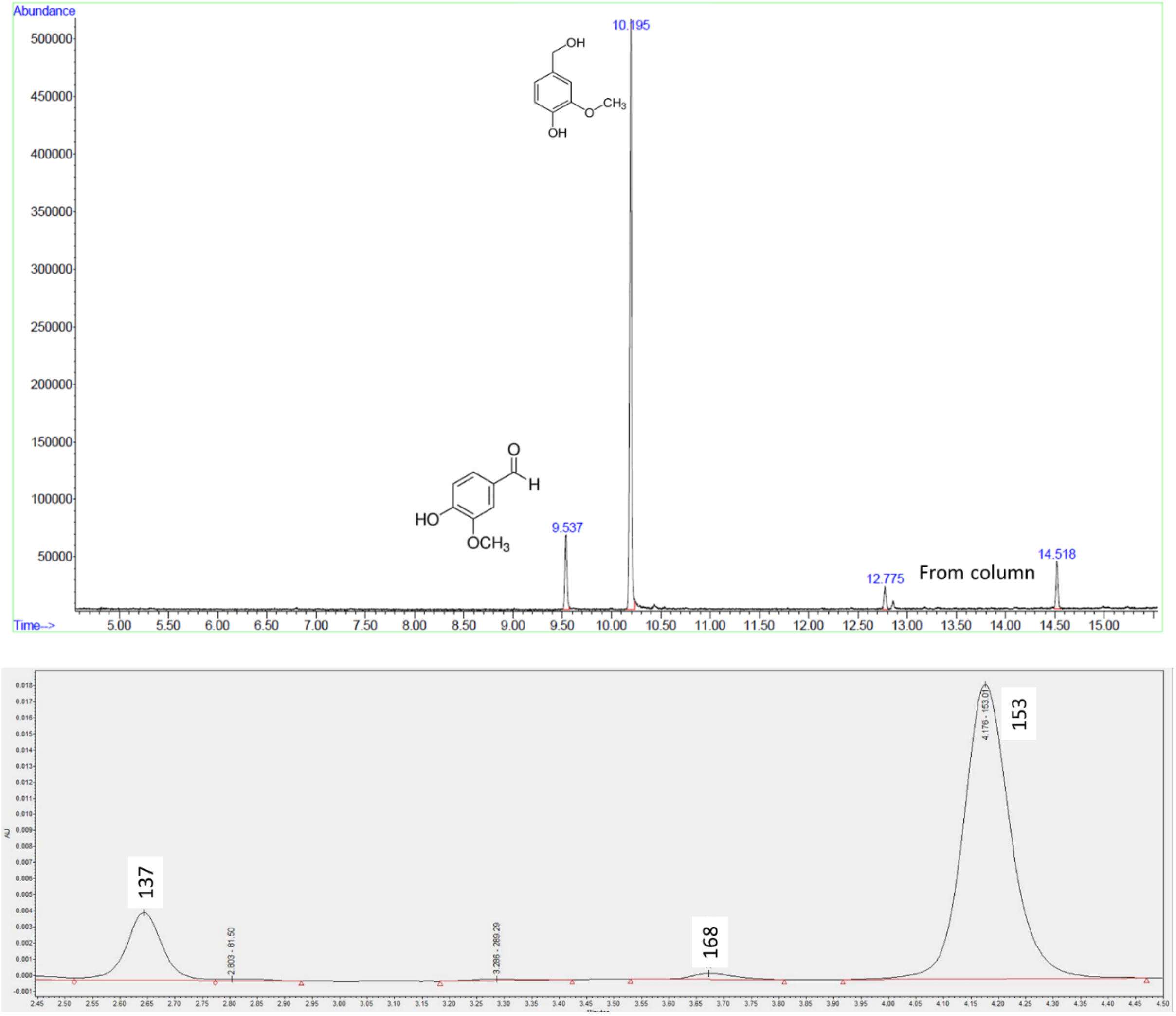

Figure S5. Oxidation products from VanOH analyzed with GC-MS and HPLC-MS.

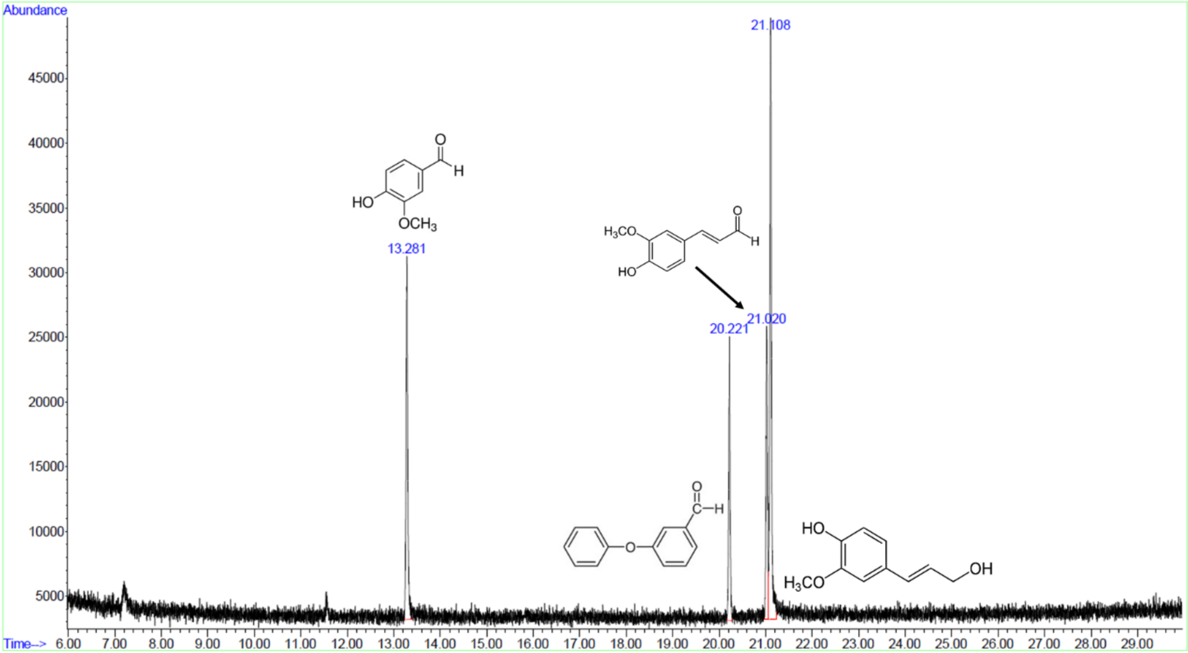

Figure S6. Oxidation products from ConOH analyzed with GC-MS.

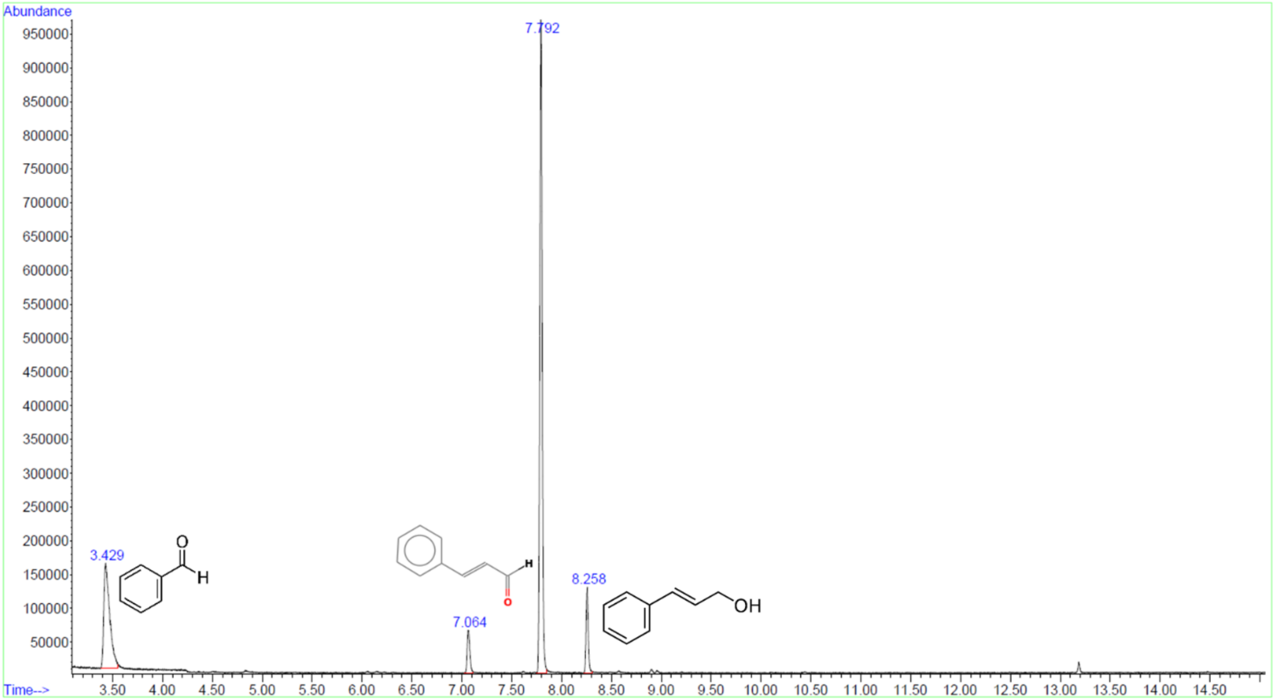

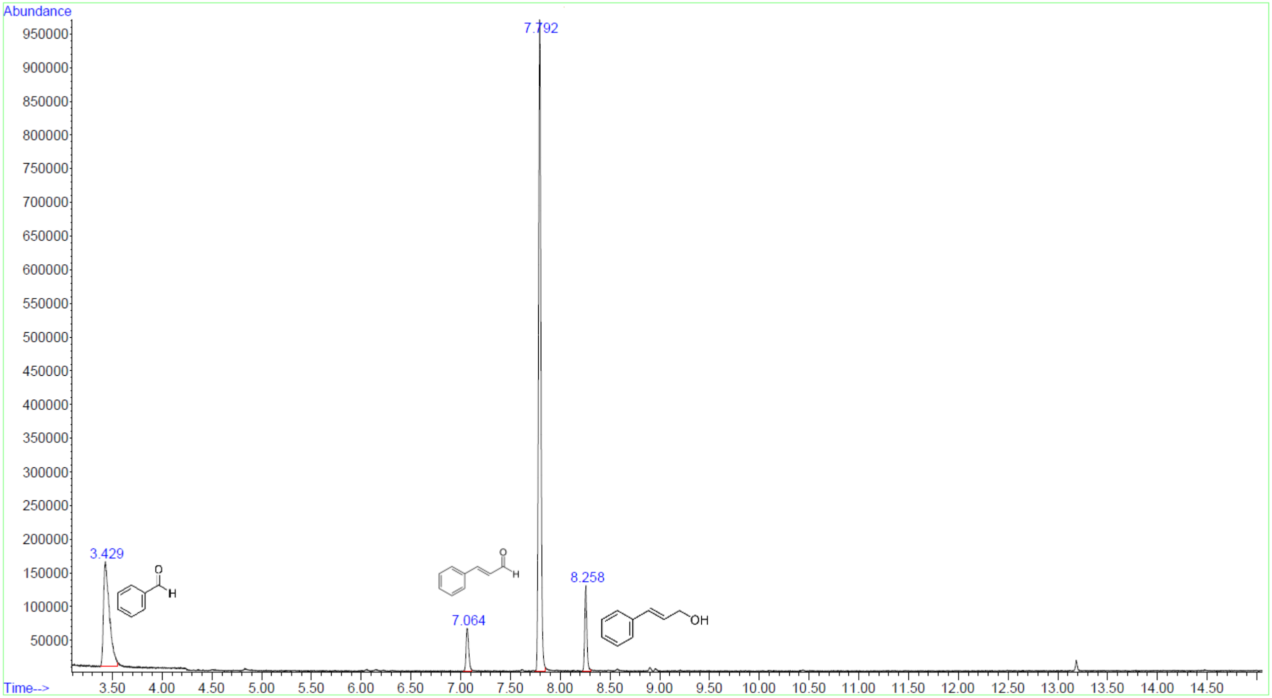

Figure S7. Oxidation products from CinOH analyzed with GC-MS.

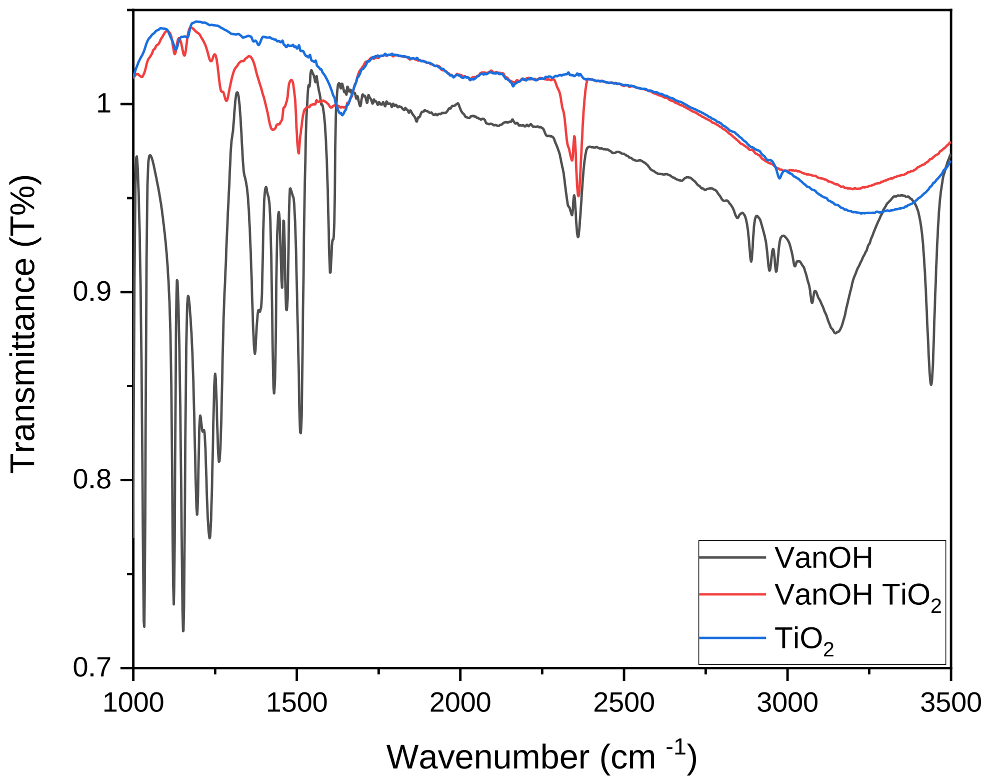
Transmittance (T%)
Wavenumber (cm -1)
VanOH
VanOH TiO2
TiO2
0.7
0.8
0.9
1
1000
1500
2000
2500
3000
3500

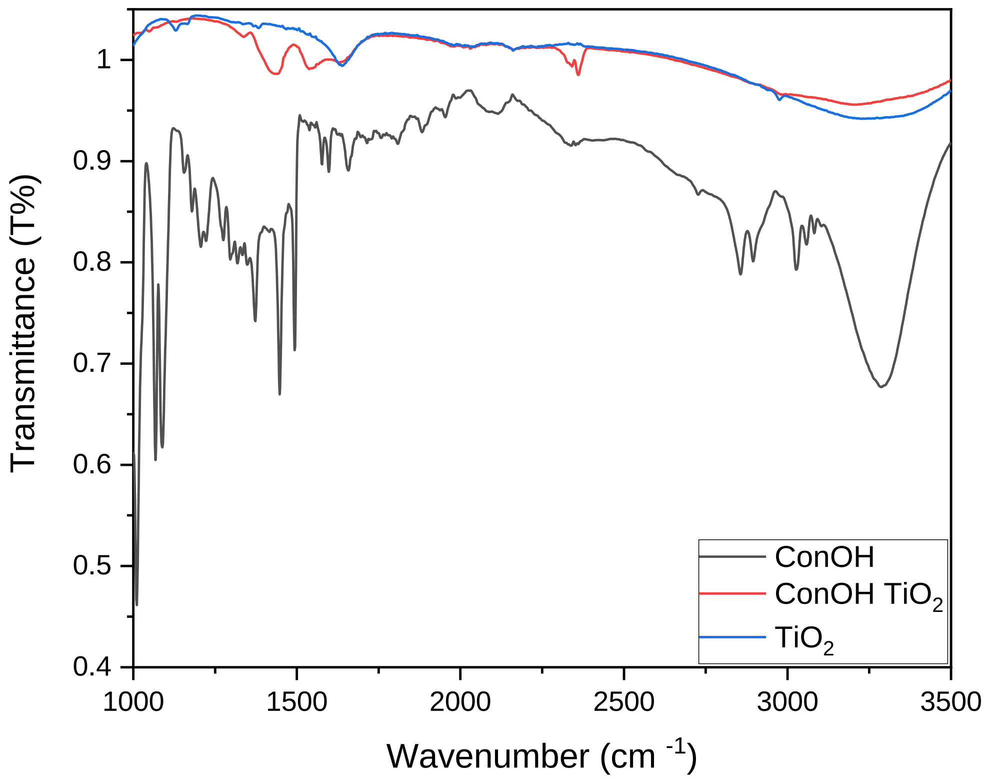
Transmittance (T%)
Wavenumber (cm -1)
ConOH
ConOH TiO2
TiO2
0.4
0.5
0.6
0.7
0.8
0.9
1
1000
1500
2000
2500
3000
3500

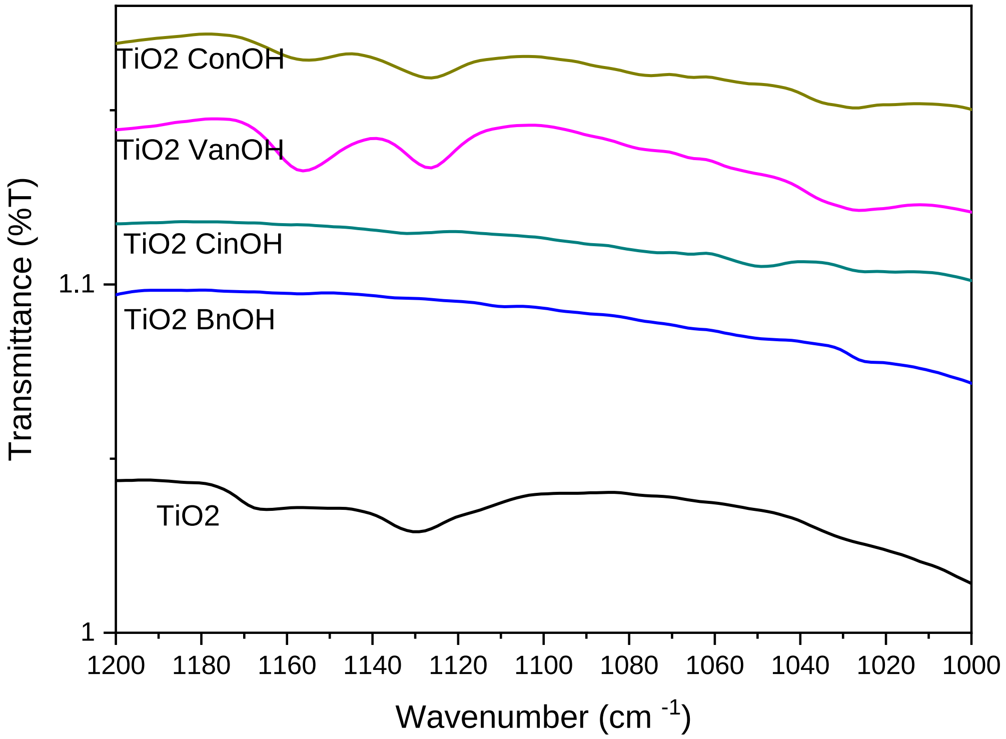
TiO2 ConOH
TiO2 VanOH
TiO2 CinOH
TiO2 BnOH
TiO2
Transmittance (%T)
1.1
1
1200 1180 1160 1140 1120 1100 1080 1060 1040 1020 1000
Wavenumber (cm -1)

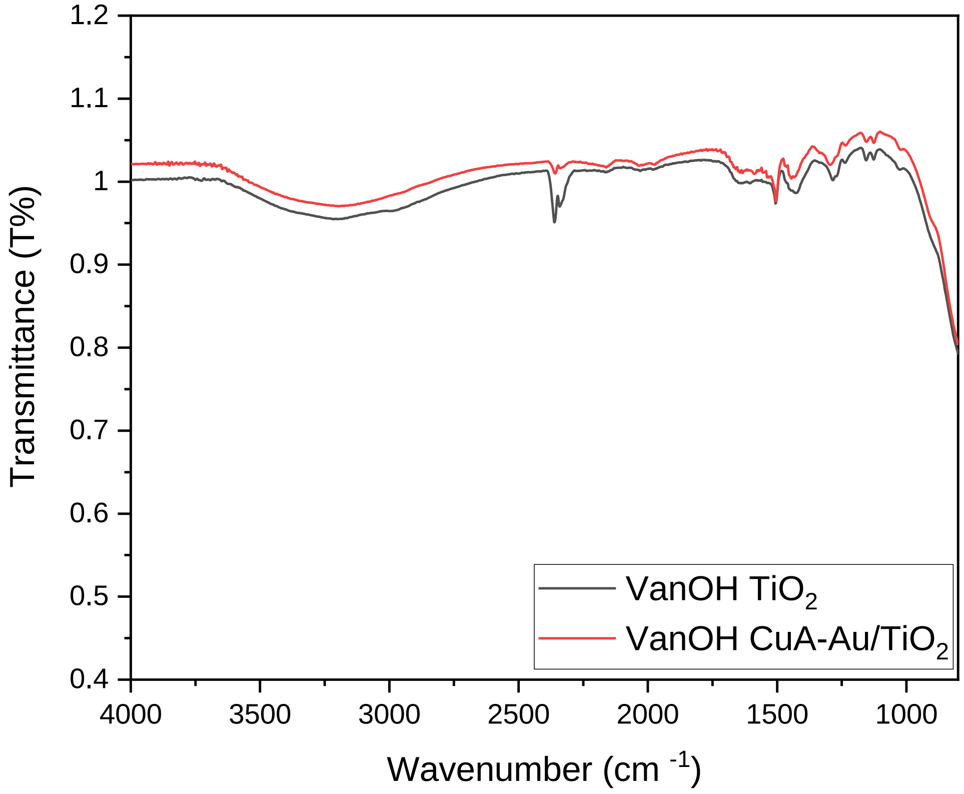
Transmittance (T%)
1.2 1.1 1 0.9 0.8 0.7 0.6 0.5 0.4
4000 3500 3000 2500 2000 1500 1000
Wavenumber (cm -1)
VanOH TiO2
VanOH CuA-Au/TiO2

Figure S8. FT-IR spectra of aromatic alcohols (BnOH, CinOH, VanOH and ConOH) adsorbed TiO_2 and VanOH adsorbed bimetallic CuA-Au-TiO_2.

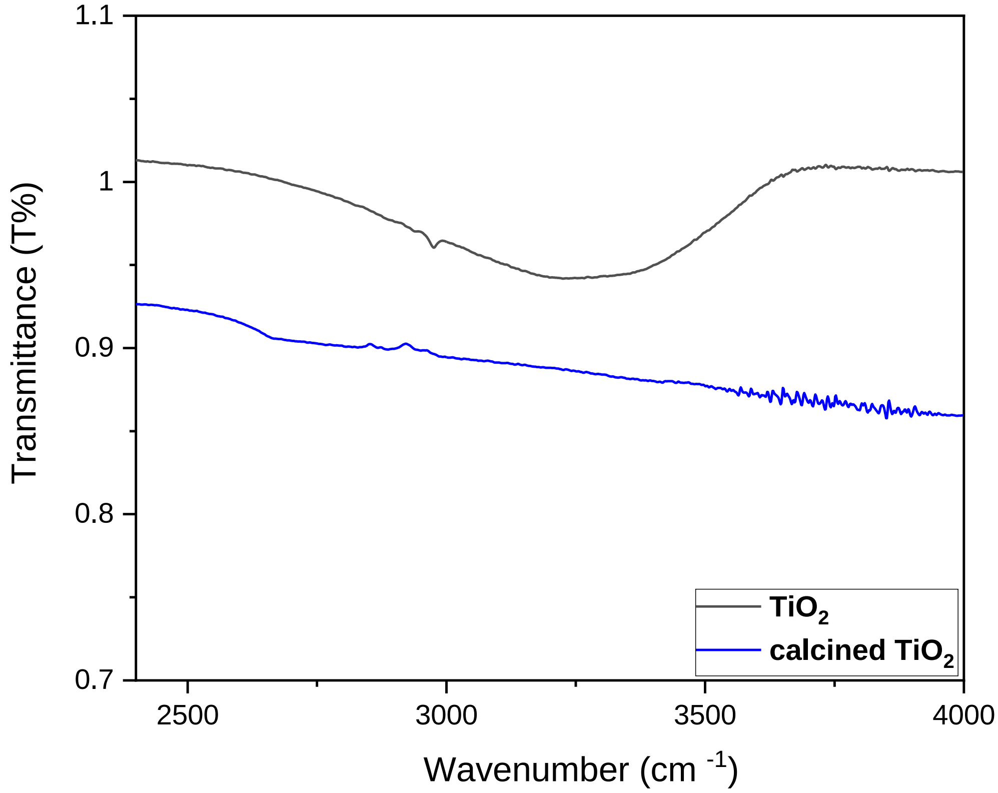

Figure S9. FT-IR spectra of sol-gel synthesized TiO_2 and calcined (600 °C) TiO_2 confirming removal of OH group.

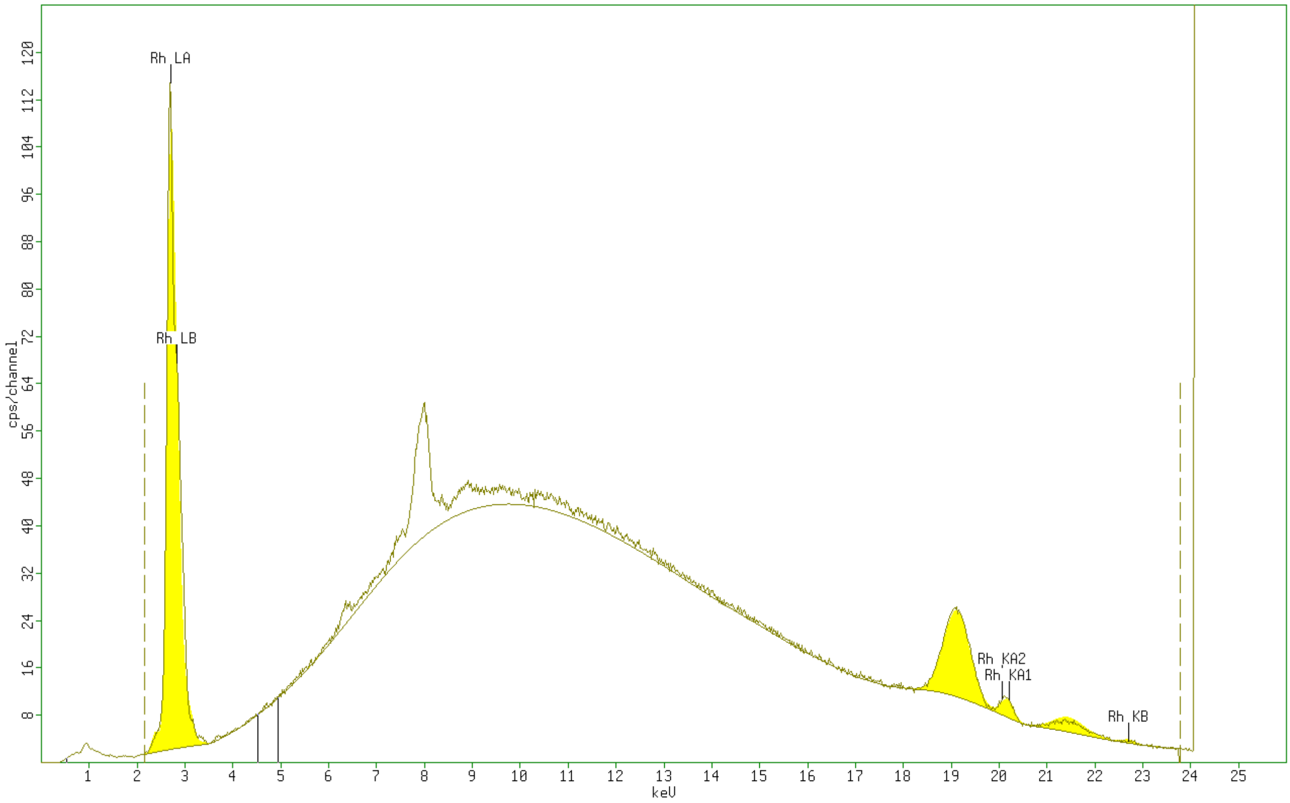

Figure S10. XRF image of CuN-/Au/-TiO_2.

Good reusability of the catalyst was demonstrated, while no metals were detected in the filtered reaction mixtures. There was no traces of Ti in liquid sample. The other observed peaks are the fingerprint from the instrument.

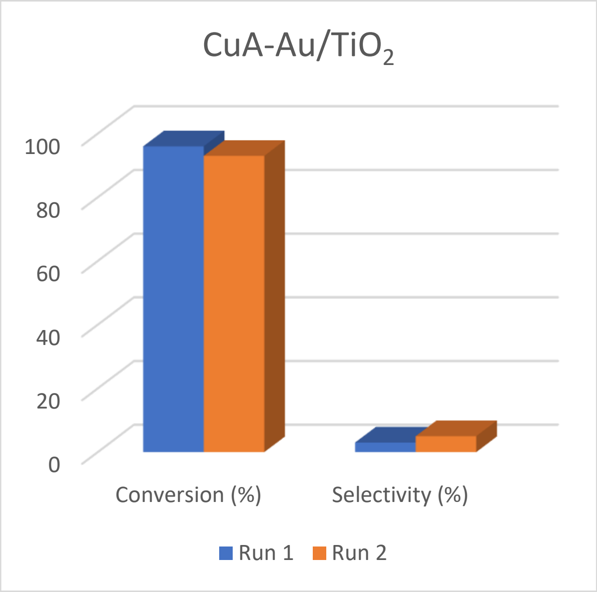

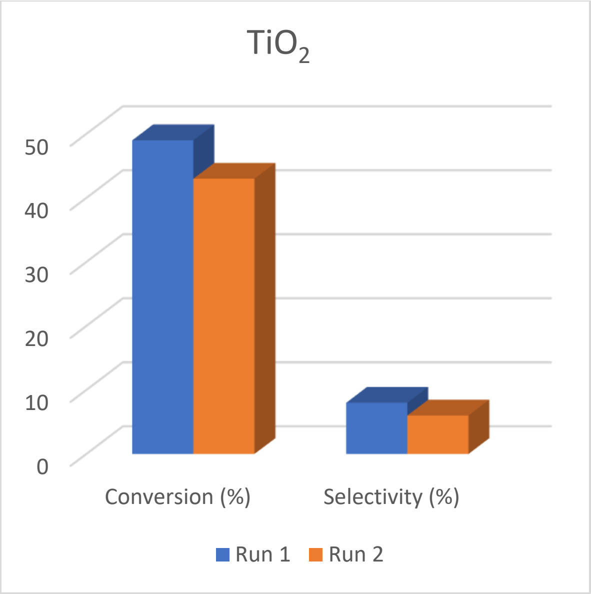

Figure S11. Photocatalytic experiment with CuA-Au/TiO_2 (left) and sol-gel synthesized TiO_2 (right) (Run 1) and after washing the catalyst with ACN and H_2O (Run2) under UV irradiation (60min) in batch system with ConOH.
